# Complementarity of modified NUTRIC score with or without C-reactive protein and subjective global assessment in predicting mortality in critically ill patients

**DOI:** 10.5935/0103-507X.20190086

**Published:** 2019

**Authors:** Manoela Lima Oliveira, Daren Keith Heyland, Flávia Moraes Silva, Estela Iraci Rabito, Mariane Rosa, Micheli da Silva Tarnowski, Daieni Fernandes, Aline Marcadenti

**Affiliations:** 1 Universidade Federal de Ciências da Saúde de Porto Alegre - Porto Alegre (RS), Brasil.; 2 Unidade de Pesquisa Clínica, Kingston General Hospital - Kingston, Ontário, Canadá.; 3 Departamento de Saúde Pública, Queen's University - Kingston, Ontário, Canadá.; 4 Departamento de Medicina Crítica, Queen's University - Kingston, Ontário, Canadá.; 5 Departamento de Nutrição, Universidade Federal de Ciências da Saúde de Porto Alegre - Porto Alegre (RS), Brasil.; 6 Programa de Pós-Graduação em Ciências da Nutrição, Universidade Federal de Ciências da Saúde de Porto Alegre - Porto Alegre (RS), Brasil.; 7 Programa de Pós-Graduação em Alimentos e Nutrição, Universidade Federal do Paraná - Curitiba (PR), Brasil.; 8 Serviço de Nutrição, Santa Casa de Misericórdia de Porto Alegre - Porto Alegre (RS), Brasil.; 9 Instituto de Pesquisa, HCor-Hospital do Coração - São Paulo (SP), Brasil.; 10 Programa de Pós-Graduação em Ciências da Saúde: Cardiologia, Instituto de Cardiologia do Rio Grande do Sul - Porto Alegre (RS), Brasil.

**Keywords:** Nutritional assessment, Nutrition, Screening, Critical care, Inflammation, Mortality, Intensive care units

## Abstract

**Objective:**

To evaluate the concordance between the modified NUTRIC and NUTRIC with C-reactive protein instruments in identifying nutritional risk patients and predicting mortality in critically ill patients. The risk of death in patient groups was also investigated according to nutritional risk and malnutrition detected by subjective global assessment.

**Methods:**

A cohort study of patients admitted to an intensive care unit. Nutritional risk was assessed by modified NUTRIC and a version of NUTRIC with C-reactive protein. Subjective global assessment was applied to diagnose malnutrition. Kappa statistics were calculated, and an ROC curve was constructed considering modified NUTRIC as a reference. The predictive validity was assessed considering mortality in 28 days (whether in the intensive care unit or after discharge) as the outcome.

**Results:**

A total of 130 patients were studied (63.05 ± 16.46 years, 53.8% males). According to NUTRIC with C-reactive protein, 34.4% were classified as having a high score, while 28.5% of patients had this classification with modified NUTRIC. According to SGA 48.1% of patients were malnourished. There was excellent agreement between modified NUTRIC and NUTRIC with C-reactive protein (Kappa = 0.88, p < 0.001). The area under the ROC curve was equal to 0.942 (0.881 - 1.000) for NUTRIC with C-reactive protein. The risk of death within 28 days was increased in patients with high modified NUTRIC (HR = 1.827; 95%CI 1.029 - 3.244; p = 0.040) and NUTRIC with C-reactive protein (HR = 2.685; 95%CI 1.423 - 5.064; p = 0.002) scores. A high risk of death was observed in patients with high nutritional risk and malnutrition, independent of the version of the NUTRIC score applied.

**Conclusion:**

An excellent agreement between modified NUTRIC and NUTRIC with C-reactive protein was observed. In addition, combining NUTRIC and subjective global assessment may increase the accuracy of predicting mortality in critically ill patients.

## INTRODUCTION

The prevalence of malnutrition is high in hospitalized patient populations and higher in populations of critically ill patients admitted to an intensive care unit (ICU),^(1,2)^ resulting in increased mortality, decreased quality of life, increased length of hospital stay and higher hospital costs.^(3-5)^ A critical illness usually has a catabolic state that manifests with an inflammatory response due to complications related to infections, multiorgan dysfunction and prolonged hospitalization time.^(6)^ C-reactive protein (CRP) is frequently measured in an ICU to evaluate inflammatory status, and it may have an important diagnostic and prognostic significance in individuals with or without infection;^(7,8)^ however, there are limitations regarding the use of CRP for monitoring critical patients, since its values may be low or normal in the first 12 hours of onset of febrile illness.^(8)^

The nutritional screening and evaluation of the critical patient is complex because many patients are on mechanical ventilation (MV) or present neurological alterations, making it difficult to collect essential information such as current anthropometric data, weight history, gastrointestinal alterations and food intake.^(9,10)^ Therefore, Heyland et al.^(11)^ developed a tool to identify patients who would benefit most from the optimization of nutritional therapy, the Nutrition Risk in the Critically Ill (NUTRIC) score. In the original version of this tool, interleukin-6 (IL-6) values were used as an inflammatory marker associated with nutritional risk. However, due to the difficulty of obtaining IL-6 in clinical practice, the NUTRIC score was later validated without the use of IL-6, yielding the modified NUTRIC (mNUTRIC) score.^(12)^ Regarding available tools for nutrition assessment in an ICU, the validity of subjective global assessment (SGA) for critically ill patients has been confirmed by recent studies.^(13-15)^

The use of CRP to replace IL-6 would be a more feasible alternative for the assessment of risk according to the NUTRIC score. A research group studied two variants of this tool in patients with assisted MV: mNUTRIC without IL-6 and NUTRIC with CRP as the inflammatory marker (NUTRIC-CRP). In this single center trial from Argentina, both variants of the NUTRIC score showed similar results to the original NUTRIC score, and the inclusion of the CRP improved the score performance in predicting mortality.^(16)^ However, in the original study for the development of the NUTRIC score, Heyland et al. did not demonstrate any incremental value to adding CRP instead of IL-6 to the original NUTRIC score.^(11)^

The use of CRP as an inflammatory marker of the NUTRIC score was rarely investigated until this moment. In a recent study, patients classified with nutritional risk (NUTRIC score 4) and severe malnourishment according to SGA showed a 28-day mortality risk seven times higher than that of patients with a NUTRIC < 4 independent of the SGA classification.^(17)^

The objective of this study was to evaluate the concordance mNUTRIC and NUTRIC-CRP instruments in identifying nutritional risk in critically ill patients and predicting clinical outcomes. Furthermore, we also evaluated the risk of death in patient groups according to nutritional risk and malnutrition diagnosis detected by SGA combined.

## METHODS

A longitudinal study was performed in a generally mixed (chirurgical, clinical and nontraumatic) 20-bed ICU of a tertiary hospital in Porto Alegre (RS), Brazil, between May 2015 and August 2016. The study included patients older than 18 years, with data collection occurring at 48 hours after admission to the ICU and whose information that was needed to complete the NUTRIC score was available in electronic medical records. Patients who were at imminent risk of death, stayed less than 24 hours in the ICU, or were admitted for elective surgeries or for overdose of illicit drugs were excluded from the study.

Information on age, sex, primary diagnosis of hospital admission, comorbidities, prescribed diet in the hospitalization unit and at the time of admission to the ICU, days of hospitalization prior to ICU admission, Sequential Organ Failure Assessment (SOFA), Acute Physiology Health Disease Classification System II (APACHE II), and CRP (mg/dL; Advia Chemistry Wide Range C-Reactive Protein - WrcCRP, Siemens Healthcare Diagnostics, Inc^®^) at the time of admission to the ICU were collected via electronic medical records. Institutional protocols were used for dietary progression based on the American Society of Parenteral and Enteral Nutrition (ASPEN) guidelines.^(18)^

For the categorization of nutritional risk, two variations of the NUTRIC tool were used: the mNUTRIC (without an inflammatory marker) and one (adapted from Moretti et al.^(16)^) including CRP values (NUTRIC-CRP). For NUTRIC-CRP scores, a different categorization of CRP from that of Moretti et al.^(16)^ was used, given that the CRP values in our study ranged from 3.5 to 402.7mg/dL. Thus, we categorized CRP in terciles to perform our analysis, and our cut-off points were < 68, 68 to 167, and ≥ 167mg/dL. Table 1 shows the variables and the scoring intervals for completing both mNUTRIC and NUTRIC-CRP instruments as well as the classification of the nutritional risk according to the final scores.

**Table 1:** Modified Nutrition Risk in the Critically Ill and Nutrition Risk in the Critically Ill with C-reactive protein score variables and classification of the nutritional risk categories

Variables	Scores			
	0	1	2	3
Age (years)	≤ 49	50 - 74	≥ 75	
APACHE II (points)	≤ 14	15 - 19	20 - 28	≥ 29
SOFA (points)	≤ 5	6 - 9	≥ 10	
Number of comorbidities	≤ 1	≥ 2		
Days of hospitalization prior to ICU admission	0	≥ 1		
CRP (mg/dL)	< 68	68 - 167	≥ 167	
mNUTRIC (without CRP)				
Low score	0 - 4 points			
High score	5 - 9 points			
NUTRIC-CRP (with CRP)				
Low score	0 - 5 points			
High score	6 - 11 points			

APACHE II - Acute Physiology and Chronic Health Disease Classification System II; SOFA - Sequential Organ Failure Assessment; ICU - intensive care unit; CRP - C-reactive protein; mNUTRIC - modified Nutrition Risk in the Critically Ill; NUTRIC-CRP - Nutrition Risk in the Critically Ill with C-reactive protein.

Subjective global assessment was performed with patients or according to information provided by their relatives, and nutritional status was classified as well-nourished (SGA A), moderately malnourished (SGA B) or severely malnourished (SGA C);^(19)^ patients with malnutrition (SGA B and C) were grouped for data analysis.

Secondary outcomes (length of ICU stay and mortality in 28 days - in ICU or after discharge) were collected in the electronic medical record or by telephone calls.

The sample size calculation was performed using the WinPepi program for Windows. Assuming an agreement of at least 70% for identification of nutritional risk according to mNUTRIC and NUTRIC-CRP scores (primary outcome), a minimum Kappa test value of 0.85, 80% power and adopting a 5% level of significance, obtaining data from at least 95 patients would be necessary.

### Statistical analysis

Statistical analysis was performed with the statistical package Statistical Package for Social Science (SPSS) version 17.0 for Windows. Categorical variables were described in percentages; continuous variables were described as the mean ± standard deviation - parametric variables - or medians (interquartile ranges) for nonparametric variables. Comparisons between patients with high and low nutritional risk according to NUTRIC-CRP/mNUTRIC scores were performed by Student's t, Wilcoxon-Mann-Whitney or Pearson's Chi-square tests. The agreement between the mNUTRIC and NUTRIC-CRP instruments was identified using the Kappa test. The sensibility, specificity, positive and negative predictive values were calculated according to habitual mathematic formulas, as well as the area under the ROC curve (AUC). Patients were grouped according to nutritional risk (mNUTRIC) and malnutrition diagnosis (SGA) into four categories: low nutritional risk and well-nourished, low nutritional risk and malnourished, high nutritional risk and well-nourished and high nutritional risk and malnourished. The associations between these tools and death within 28 days (exploratory analysis) were evaluated using Cox regression, adjusted for sex. A 5% significance level was considered.

The project is in line with Brazilian Resolution (CNS No. 466/12), approved by the Institutional Ethics Committee of the *Universidade Federal de Ciências da Saúde de Porto Alegre* under the record number 1,073,256 and by the Ethics Committee of the *Irmandade da Santa Casa de Porto Alegre* under the record number 1,030,523. The study was also conducted in accordance with the Helsinki Declaration.

## RESULTS

A total of 130 patients were included in the current study; their characteristics are described in table 2. Regarding the main reason for hospital admission prior to admission to the ICU, 35.4% were related to gastrointestinal diseases; 20.8% were related to pneumological, neurological or oncological diseases; 15.4% were related to cardiac, nephrological or endocrine diseases; and 3.8% were related to sepsis, shock or postoperative complications. The median length of hospital stay prior to ICU admission was 5.0 (1.0 - 18.0) days.

**Table 2:** Characterization of the sample according to the Modified Nutrition Risk in the Critically Ill and Nutrition Risk in the Critically Ill with C-reactive protein scores

Variables	All sample (n = 130)	mNUTRIC score			NUTRIC-CRP score		
		Low score (n = 93)	High score (n = 37)	p value	Low score (n = 59)	High score (n = 31)	p value
Age (years)	63.05 ± 16.46	60.2 ± 16.6	69.5 ± 13.4	0.003*	60.2 ± 16.5	70.2 ± 14.1	0.005*
Males	53.8	55.9	48.6	0.40†	57.6	48.4	0.40†
APACHE II	6.0 (4.5 - 20.0)	6.0 (3.0 - 12.0)	26.0 (20.0 - 32.0)	< 0.001‡	6.0 (3.0 - 12.0)	26.0 (20.0 - 32.0)	< 0.001‡
SOFA	0.0 (0.0 - 6.3)	0.0 (0.0 - 2.0)	10.0 (7.0 - 11.0)	< 0.001‡	0.0 (0.0 - 2.0)	10.0 (7.0 - 11.0)	< 0.001‡
mNUTRIC (points)	3.0 (2.0 - 5.0)	3.0 (2.0 - 3.0)	7.0 (6.0 - 8.0)	< 0.001‡	3.0 (2.0 - 3.0)	7.0 (6.0 - 8.0)	< 0.001‡
NUTRIC- CRP (points)	5.16 ± 2.37	4.0 (3.0 - 5.0)	8.0 (6.8 - 9.0)	< 0.001*	3.7 ± 1.1	7.9 ± 1.6	< 0.001*
CRP (mg/dL)	112.3 (41.4 - 198.0)	100.7 (33.2 - 174.6)	130.0 (52.5 - 236.3)	0.159‡	92.3 (31.6 - 153.0)	179.0 (67.7 - 240.0)	0.009‡
Malnutrition	48.1	41.9	64.9	0.021†	47.5	51.6	0.708†
Mechanical ventilation	60.6	51.6	83.8	0.005†	57.6	87.1	0.005†
Days of hospital stay prior to ICU admission	5.0 (1.0 - 17.5)	6.0 (1.0 - 18.0)	5.0 (1.0 - 16.0)	0.858‡	10.0 (2.0 - 26.0)	3.0 (1.0 - 13.0)	0.016‡
Days of ICU stay	8.0 (3.0 - 16.0)	7.0 (3.0 - 14.0)	9.0 (5.0 - 22.0)	0.042‡	10.0 (5.0 - 16.0)	8.0 (5.0 - 22.0)	0.51‡
Death in the ICU	38.9	29.0	64.9	< 0.001†	23 (39.0)	21 (67.7)	0.009†
Death within 28 days	34.8	28.9	54.1	0.007†	21 (36.2)	18 (58.1)	0.048†

mNUTRIC - modified Nutrition Risk in the Critically Ill; NUTRIC-CRP - Nutrition Risk in the Critically Ill with C-reactive protein; APACHE II - Acute Physiology and Chronic Health Disease Classification System II; SOFA Sequential Organ Failure Assessment; CRP - C-reactive protein; ICU - intensive care unit.

* Student's t test;

† Pearson's Chi-square test;

‡ Wilcoxon-Mann-Whitney test. Results expressed as mean ± standard deviation, %, median (interquartile range) or n (%).

The main causes of ICU admission were postoperative complications (33.1%), septic shock or worsening of overall conditions (21.5%), infection or sepsis (12.3%), heart problems (11.5%), and gastrointestinal or pneumological complications (10%). Of the postoperative complications, 51.2% were admitted to the hospital due to gastrointestinal diseases; 11.6% were admitted due to pneumological, neurological or oncological diseases; 18.6% were admitted due to cardiac, nephrological or endocrine diseases; and 2.3% were admitted due to postoperative complications after discharge. Of the second largest group, 27.6% were admitted to the hospital due to gastrointestinal diseases; 31% were admitted due to pneumological, neurological or oncological diseases; 17.2% were admitted due to cardiac, nephrological or endocrine diseases; and 6.9% were admitted due to sepsis, shock or postoperative complications after discharge.

According to dietary prescription in the ICU, 44.6% were on an oral diet, 53.8% on an enteral diet and 10% on a parenteral diet. Regarding multiple routes of feeding, 5 individuals were on oral plus enteral nutrition, 1 on enteral plus parenteral nutrition and 2 on the three routes simultaneously. The median length of ICU stay was 8.0 (3.0 - 16.8) days. In total, 60.8% required MV. The mortality rate in the evaluated population was 39.2%.

Regarding the risk assessment according to mNUTRIC scores, 71.5% were classified with low scores (≤ 4 points), and 28.5% were classified with high scores (≥ 5 points). For the analysis of NUTRIC-CRP scores, the data of 90 individuals whose CRP values were available at ICU admission via electronic medical records were used. Of these, 65.6% presented a low score (≤ 5 points), and 34.4% were classified as high score (≥ 6 points). According to SGA, 48.1% of patients were classified as malnourished (categories B and C).

The agreement between the mNUTRIC and NUTRIC-CRP instruments was considered excellent (n = 90; Kappa = 0.88, p < 0.001). The sensitivity of the NUTRIC-CRP instrument (considering mNUTRIC as the reference method) was 90.3%, while the specificity was 96.6%. The positive predictive value was 93.3%, the negative predictive value was 96.6%, and the AUC was 0.942 (0.881 - 1.000).

Table 2 shows the characterization of the sample according to the categorization of mNUTRIC and NUTRIC-CPR scores (low scores and high scores). Patients with a high score of nutritional risk are older, have higher CPR levels, have APACHE and SOFA at elevated values and present a higher incidence of death in comparison to patients with a low score of nutritional risk, independent of the NUTRIC version. The frequency of malnourishment was higher in patients with high nutritional risk than in patients with low nutrition risk only when the mNUTRIC score was applied.

According to the Cox regression, adjusted for sex, patients with high nutritional risk presented a risk of death within 28 days of 2.685 times higher (95% confidence interval - 95%CI 1.423 - 5.064; p = 0.002) than patients with low nutritional risk according to their NUTRIC-CRP scores. The mNUTRIC score was also significantly associated with the incidence of death within 28 days (hazard ratio - HR = 1.827; 95%CI 1.029 - 3.244; p = 0.040).

The risk of death in the categories considering nutritional risk and malnutrition diagnosis by SGA combined was significant only in patients with a high mNUTRIC score and who were malnourished, as demonstrated in table 3. Regarding the NUTRIC-CRP score, the risk of death was also significantly increased only in patients with a high score and who were malnourished (HR = 4.112; 1.738 - 9.727).

**Table 3:** Complementarity of the modified Nutrition Risk in the Critically Ill score and subjective global assessment for predicting mortality in 28 days in critically ill patients - Cox regression adjusted for sex

Category	HR (95%CI)
Low mNUTRIC score and well-nourished (SGA A)	Reference
Low mNUTRIC score and malnourished (SGA B or C)	1.429 (0.643 - 3.178)
High mNUTRIC score well-nourished (SGA A)	1.750 (0.727 - 4.215)
High mNUTRIC score and malnourished (SGA B or C)	2.167 (1.029 - 4.563)

HR - hazard ratio; 95%CI - 95% confidence interval; mNUTRIC - modified Nutrition Risk in the Critically Ill; SGA - subjective global assessment.

## DISCUSSION

In our study, we evaluated the agreement between the mNUTRIC and NUTRIC-CRP scores, and according to the results, we suggest that the use of an inflammatory marker for evaluation/stratification of nutritional risk in an ICU may not be required. Both scores showed excellent agreement and were positively associated with mortality when combined with malnutrition diagnosed by SGA.

A similar prevalence of nutritional risk was observed in the current study when applied to the mNUTRIC and NUTRIC-CRP scores: 28.5% and 34.4%, respectively. Several studies^(20-25)^ have evaluated nutritional risk by NUTRIC in critically ill patients, and the prevalence of high scores ranges from 22.4%^(21)^ to 67.9%.^(22)^ The lower prevalence of nutritional risk in our study may be explained by the severity of patients - the APACHE II and SOFA scores were relatively low, and only 60% of the patients were mechanically ventilated.

Moretti et al.^(16)^ also compared mNUTRIC scores (without IL-6 values) and NUTRIC-CRP scores and demonstrated a lower prevalence of nutritional risk according to NUTRIC-CRP scores than mNUTRIC scores (25% and 34%, respectively), while in the current study, the opposite was observed. This may be related to the CRP cutoff points adopted for the nutritional risk stratification in that study (< or ≥ 10mg/dL); we decided to categorize CRP values in tertiles because this variable had a nonnormal distribution. In addition, critically ill patients present naturally higher values of CRP.

Both scores had satisfactory ability in predicting mortality in the current study, despite being an exploratory analysis. In fact, there is consistent literature about the positive association between a high NUTRIC score and death.^(11,12,21,25)^ It was observed in the study of NUTRIC validation that each point increase in the NUTRIC score resulted in a significant increase in the rate of mortality.^(11)^ This was also demonstrated in a study conducted by Rahman et al that validated the mNUTRIC score.^(12)^ In another study conducted with 482 patients with sepsis, the AUC of the mNUTRIC score and the original NUTRIC score for predicting 28-day mortality were 0.762 and 0.757, respectively.^(25)^

Our results suggest that the risk of death is significantly higher in patients with a high score of nutritional risk plus malnutrition diagnosis. A prospective study including 439 critically ill patients demonstrated that the discriminative value for hospital mortality was similar for high mNUTRIC scores (C- statistics = 0.66) and malnutrition (C- statistics = 0.60), while the combination of both had a significantly better discriminative ability than either of those tools alone (C- statistics = 0.70). In this study, the risk of mortality was equal to 14.43 (95%CI 5.38 - 38.78) in patients who were both malnourished and had high mNUTRIC scores.^(23)^ The predictive validity of SGA in critically ill patients was demonstrated in previous studies.^(13-15)^ The NUTRIC score is an important nutritional risk assessment tool to guide nutrition intervention in critically ill patients. Several studies have shown that the beneficial effects of nutritional support are more evident in high-risk patients.^(11,12,26)^ The international societies guidelines recommend the use of the NUTRIC score to identify critically ill patients with nutritional risk and recommend that nutritional goals should be achieved early in patients with a high NUTRIC score.^(18,27)^

This was a small single center study with a limited sample evaluated, which may be considered a limitation of the current study. However, a sample size calculation was performed to evaluate the primary outcome (concordance between both NUTRIC scores), with a positive result. It is noteworthy that the NUTRIC score has already been translated and adapted to the Portuguese language.^(28)^

## CONCLUSION

An excellent agreement between mNUTRIC and NUTRIC-CRP scores was observed, suggesting that the use of an inflammatory marker for stratification of nutritional risk in intensive care may not be required. Both scores were positively associated with mortality, and the risk of death was particularly increased in patients with a high mNUTRIC score and who were malnourished. Therefore, we conclude that the use of the NUTRIC score without inflammatory markers is feasible for use in clinical practice in critically ill patients. It should be complemented by malnutrition diagnosis with subjective global assessment to improve the accuracy of predicting outcomes.
